# Effects of upper-limb aerobic exercise plus general exercise versus general exercise alone among patients with spinal cord injury in northern Nigeria: A protocol for a randomized controlled trial

**DOI:** 10.1371/journal.pone.0321932

**Published:** 2025-05-12

**Authors:** Fatima Kachalla Gujba, Sonill Sooknunan Maharaj, Aminu Alhassan Ibrahim

**Affiliations:** 1 Department of Physiotherapy, School of Health Sciences, College of Health Sciences, University of KwaZulu-Natal, Durban, South Africa; 2 Department of Medical Rehabilitation, College of Medical Sciences, University of Maiduguri, Maiduguri, Borno State, Nigeria; 3 Physiotherapy Department, Tishk International University, Erbil, Iraq; SARAH Network of Rehabilitation Hospitals: Rede SARAH de Hospitais de Reabilitacao, BRAZIL

## Abstract

**Introduction:**

Spinal cord injury (SCI) is a devastating injury often associated with immobility, leading to numerous complications, with cardiovascular disorders being among the major causes of mortality. Upper-limb aerobic exercise (ULAE) may help to retrain and regain some of the abilities lost through SCI and minimize secondary complications. The purpose of this study is to determine the effects of ULAE plus general exercise compared to the same general exercise on cardiovascular parameters, functional independence, and quality of life among patients with SCI in northern Nigeria.

**Method:**

This is a multicenter, assessor-blind, parallel group, randomized controlled superiority trial to be conducted among SCI patients attending three tertiary hospitals in Kano State, northern Nigeria. Participants fulfilling the study criteria will be randomized into either ULAE (arm ergometer and overhead pulley exercises) plus general exercise group or general exercise only group. Both groups will receive their respective interventions three times per week for 8 weeks. The primary outcomes will be rate pressure product (calculated as systolic blood pressure x heart rate) and functional independence. The secondary outcomes will be blood pressure and quality of life. All outcomes will be assessed at baseline, 8 weeks post-intervention, and at 14weeks follow-up.

**Discussion:**

This study will be the first to determine the effects of ULAE plus general exercise versus general exercise alone among patients with SCI in northern Nigeria. The outcome of this study could provide valuable guidance for rehabilitation professionals in selecting a low-cost and effective intervention for patients with SCI, particularly in resource-constrained settings.

**Trial registration:**

Pan African Clinical Registry (PACTR202306580460880). Registered on June 12, 2023.

## Introduction

Spinal cord injury (SCI) is a devastating injury accompanied by physical, psychosocial, and economic problems, as well as significant personal and social repercussions [1]. It is often associated with distinct diverse secondary complications including acute complications such as bradyarrhythmias, hypotension, impaired temperature, pain, spasticity, and autonomic dysreflexia [2] and chronic complications such as cardiopulmonary, intestinal, urinary bladder, and sexual disorders [3]. Long-term secondary complications can increase morbidity and negatively impact functional independence, community participation, and quality of life (QOL) [3]. Additionally, individuals with SCI, depending on their type of injury, often experience reduced physical activity levels, which is considered antecedent to development of various complications [4]. Individuals with SCI are approximately 40% less physically active compared to able-bodied counterparts [5].

A major complication linked to physical inactivity in individuals with SCI is the increased prevalence and earlier onset of cardiovascular disease (CVD) [6]. Research suggests that individuals with SCI, particularly those with cervical and thoracic levels of injury, often struggle with unstable blood pressure (BP), leading to persistent hypotension and episodes of uncontrolled hypertension [7].

Following SCI, there is an immediate disruption in connections between supraspinal structures and sympathetic preganglionic neurons. This leads to instability, causing spinal circuits to solely regulate sympathetic activity, which impacts BP control. Acute SCI, particularly at the cervical level, often results in severe hypotension and persistent bradycardia, contributing to neurogenic shock. However, the recognition and management of these cardiovascular dysfunctions following SCI pose significant clinical challenges. Moreover, CVD in both the acute and chronic stages of SCI is among the most common causes of mortality in this population [8].

As a greater number of individuals with SCI experience a significant reduction in daily physical activity [9], which increases the risk of secondary health issues and comorbidities post-injury [2,3,10], addressing physical inactivity is a top health care priority [11]. Although exercises such as strengthening and stretching are commonly employed in SCI rehabilitation to improve strength and flexibility, respectively, these exercises are insufficient to address cardiorespiratory fitness. However, one critical intervention to maintain and improve cardiorespiratory fitness and cardiometabolic health, while enhancing upper body strength and endurance, is upper-limb aerobic exercise (ULAE) [12]. Indeed, our Delphi study [unpublished] aimed at identifying the most effective exercise interventions for individuals with SCI in northern Nigeria, suggested a combined arm ergometer and reciprocal pulley exercise program as the most appropriate and cost-effective intervention to improve cardiorespiratory function and functional independence for this population. Moreover, one advantage of this proposed exercise program is that the equipment (arm ergometer and reciprocal pulley) can be improvised and adapted for home use, which is particularly important in resource-constrained settings.

Previous trials evaluating the effects of ULAE for patients with SCI demonstrated promising results in terms of improved pulmonary function [13,14] though failed to show significant effects on functional status, QOL, psychological state, level of disability, and metabolic syndrome [13,14,15], which could be attributed to inadequate sample size and lack of follow-up. Additionally, to our knowledge, no randomized controlled trial (RCT) has been conducted to evaluate the impact of any ULAE program for individuals with SCI in northern Nigeria. Since an ULAE program has been suggested following our Delphi study (unpublished), it is crucial to evaluate its effectiveness to provide recommendations for rehabilitation professionals in our context and beyond. Thus, the purpose of this study is to determine the effects of ULAE plus general exercise program compared to the same general exercise among patients with SCI in northern Nigeria.

We anticipate that ULAE (arm ergometer and reciprocal pulley exercises) combined with a general exercise focusing on strength and flexibility, will significantly improve cardiorespiratory fitness and cardiometabolic health. By enhancing these parameters, the intervention may also contribute to better functional independence, improved QOL, and reduced risk of secondary complications. This could ultimately lead to a reduction in morbidity and mortality rates among this population.

## Methods

### Ethical consideration

Ethical approval was obtained from the ethics committees of Aminu Kano Teaching Hospital (AKTH; Ref: NHREC/28/01/2020/AKTH/EC/3013), Kano State Ministry of Health (Ref: NHREC/17/03/2018), and National Orthopaedic Hospital Dala (NOHD; Ref: NOHD/RET/43). Any protocol amendments will be communicated to the various ethics committee and updated in the Pan African Clinical Trials Registry.

### Objectives

The primary objective is to determine the effects of an 8-week ULAE plus general exercise versus general exercise alone on rate pressure product (RPP) and functional independence among patients with SCI. The secondary objective is to determine the effects of these interventions on blood pressure (BP) (systolic blood pressure [SBP] and diastolic blood pressure [DBP] and QOL.

### Hypotheses

The primary hypothesis is that participants receiving ULAE plus general exercise will demonstrate greater improvements in RPP and functional independence compared to those receiving general exercise alone at 8 weeks post-intervention and at the 14 weeks follow-up. The secondary hypothesis is that participants receiving ULAE plus general exercise will demonstrate greater improvements in BP and QOL compared to those receiving general exercise alone at 8 weeks post-intervention and at the 14 weeks follow-up.

### Study design

This is a multi-center, assessor-blind, parallel group, randomized controlled superiority trial to be conducted among patients with SCI in northern Nigeria. The trial is designed in accordance with the Consolidated Standards of Reporting Trials (CONSORT) guidelines and reported according to the Standard Protocol Items: Recommendations for Interventional Trials (SPIRIT) checklist [16] (see S1 Appendix).

### Study settings

The study is being conducted in the outpatient unit of the Physiotherapy Department at each of the three major healthcare facilities in Kano State, northern Nigeria: AKTH, Murtala Mohammed Specialist Hospital (MMSH), and NOHD.

### Participants

**Fig 1:**
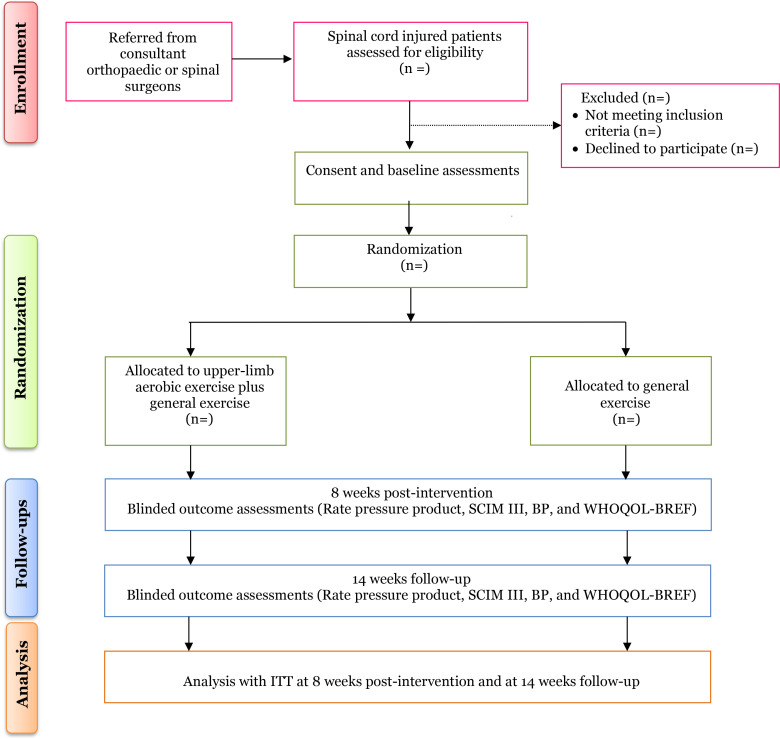
Flow of participants through the study.

Potential study participants are individuals with SCI who will be identified by consultant spine surgeons and subsequently referred to the physiotherapy outpatient unit. The study flow is illustrated in Fig 1

### Inclusion criteria

To be eligible for the study, participants must meet the following criteria:

1. Received medical clearance from their physicians confirming they are fit to participate in exercise.
2. Male or female aged 18–65 years old.
3. Diagnosed with traumatic SCI between the 7^th^ cervical (C7) and 5^th^ lumbar (L5) vertebrae, with a minimum of 3 months post-injury.
4. Upper limb muscle power of at least 3, as assessed by gross muscle power testing, to be able to propel an arm crank ergometer.
5. Willing to participate and having provided signed informed consent.

### Exclusion criteria

Participants will be excluded from the study if they meet any of the following conditions:

1. SCI level other than C7 to L5.
2. Presence of serious or unstable cardiovascular or neurological disorders.
3. Brain injury due to trauma.
4. Other secondary health issues (e.g., pressure sores, bladder infections) and medical conditions (e.g., severe psychiatric disease) that prevent participation in physical activities.

### Informed consent

Informed consent will be obtained by the physiotherapist responsible for eligibility assessment after providing participants with a detailed explanation of the study, including its benefits and procedures. Participants will be informed that they have the right to withdraw from the study at any time without any prejudice or negative consequences.

### Sample size calculation

To our knowledge, no previous study has evaluated the effects of ULAE plus general exercise program compared to general exercise alone among SCI patients, using the RPP (product of SBP and HR) as an outcome. However, since the minimal clinically important difference (MCID) for total Spinal Cord Independence Measure III (SCIM III) has been reported to be 10.0 points [17], this study was powered to detect a minimum difference of 10% points between the ULEA group and control group (assuming a standard deviation [SD] of 12.0, based on the study by Akkurt et al. [15]. Using the following parameters— F-test, repeated measures, between-subjects analysis of variance (ANOVA); alpha (*α*) level of 0.05; power of 0.80; effect size of 0.40 when accounting for 3 repeated measures; and correlation among repeated measures of 0.5— a total of 36 participants was required. Accounting for a potential dropout rate of 30% (n = 10), the final sample size was determined to be 46 participants. Calculations were performed using G Power 3.1.9.2 software [18].

### Randomization and blinding

After ensuring study eligibility and completing baseline assessments, the participants will be randomly assigned to either ULAE plus general exercise group or general exercise alone (control) group. Randomization will be performed by an independent person (a record officer) at each study site using computer-generated random numbers. To ensure allocation concealment, sequentially numbered, opaque, sealed envelopes will be prepared in accordance with the generated random sequence. Upon the availability of an eligible participant at the scheduled appointment, the next envelope will be opened to determine the intervention group to which the participant is allocated. Outcome assessors will be blinded to group allocation. However, owing to the nature of the study interventions, blinding of the physiotherapists administering treatment will not be possible. Unbinding will only be permissible in cases where a participant experiences a serious adverse event or after the study’s completion.

### Explanation for the choice of comparators

General exercises are typically prescribed by physiotherapists for individuals with SCI, as they enhance cardiovascular health, muscle strength, and flexibility; prevents secondary complications; improve mood and mental well-being; and enhance overall QOL. As such general exercise may be considered a standard of care. This study intends to determine the effects of adding ULAE to general exercise (i.e., the experimental intervention) compared to general exercise alone (i.e., active control intervention). The use of an active control group will help to minimize the likelihood of attributing improvements observed in the experimental group solely to a placebo effect.

### Study interventions

All the study interventions will be administered under the supervision of physiotherapists holding a postgraduate degree in musculoskeletal physiotherapy. They will receive training on the study intervention protocols prior to the commencement of the study by the primary researcher. Both groups will receive general exercise, while participants in the ULAE group will additionally perform arm ergometer and overhead pulley exercises. All interventions will be administered three times per week for 8 weeks.

### Upper-limb aerobic exercise (ULAE)

A Bike Portable Under-Desk Arm Leg Foot Exerciser Stationary (TDC T-C CA, Japan) with an adjustable multi-level resistance system will be used to perform ULAE, based on the recommendations of the American College of Sports Medicine (ACSM) [19]. The exercise protocol will consist of three phases: warm-up, exercise, and cool-down. For both the warm-up and cool-down phase, participant will pedal the ergometer for 5 minutes without resistance. The exercise phase will be tailored according to each participant’s ability and strength, and will involve 20 minutes of pedaling aiming for light to moderate intensity. The target heart rate will be maintained between 50–70% of estimated maximum heart rate (220 – age). Participants will be encouraged to maintain a pedaling rate between 50 and 60 revolutions per minute (RPM). Resistance will be gradually increased over the weeks by adjusting the tension knob on the exerciser. The heart rate of the participants will be monitored using a digital heart rate monitor to be worn on their wrists. Following the arm ergometer exercise and adequate rest, participants will perform reciprocal or overhead pulley exercise using an 85-inch reciprocal pulley (Samvsine, USA) for 10 minutes. The exercise sessions will be conducted three times per week on non-consecutive days for 8 weeks.

### General exercise

The general exercise program will be tailored to the patient’s neurological level and abilities, and will include passive, active-assisted, and free active range of motion (ROM) exercises, as well as stretching and strengthening exercises for the upper and lower body [15]. These exercises will be performed in various positions including sitting, prone, supine, quadruped, kneeling, and standing. ROM exercises will utilize the reciprocal pulley while strengthening exercises will involve the use of sandbags and dumbbells of varying weights. The exercise will be targeted at key muscles of body (i.e., deltoids, triceps, biceps, pectoralis, wrist flexors, wrist extensors, quadriceps, hamstrings, and gastrocnemius). Exercises will be performed at moderate intensity, which is considered safe for SCI people, as recommended by the ACSM [19]. Each exercise will be held for 10–15 seconds, repeated 10 times , with a 5-second rest between repetitions and a 60-second rest between different exercise types. The exercise sessions will be conducted three times per week for 8 weeks.

### Strategies to improve adherence to interventions

To enhance adherence to the interventions, participants will be thoroughly briefed during the consent process on the importance of attending all treatment sessions and completing outcome assessments after intervention and at the 14 weeks follow-up. Additionally, participants will receive regular follow-up reminders via phone calls to encourage consistent participation.

### Relevant concomitant care permitted or prohibited during trial

Relevant concomitant care, such as medications, will be permitted during the trial, However, any other experimental treatments that could confound the effects of the studied interventions will be prohibited.

### Adverse events

All necessary precautions will be taken to prevent the occurrence of any adverse events. Participants will be fully informed of the potential for both serious and non-serious adverse events prior to the commencement of the study interventions. In the event of a serious adverse event (e.g., injuries, persistent excruciating pain, decrease in spine ROM) at any point during the trial, the primary researcher will be immediately notified. The participant may be withdrawn from the study, and appropriate medical care will be sought from a physician. Additionally, reports of serious adverse events will be submitted to the Health Research Ethics Committees of NOHD, AKTH, and Kano State Ministry of Health, Nigeria. Non-serious adverse events that may occur include physical exertion, muscle soreness, dizziness, and exacerbation of muscle or joint pain. These will be monitored and managed appropriately throughout the trial.

### Baseline data collection

Sociodemographic and clinical characteristics, including age, sex, marital status, education level, occupational status, height, weight, body mass index (BMI), duration of injury, type of injury, level of affectation, and presence of comorbidities will be collected. American Spinal Injury Association (ASIA) Impairment Scale will be used to classify the level of injury [20]. Each participant will be assigned a unique ID for anonymity and tracking purposes.

### Data collection tools and outcome measures

**Fig 2:**
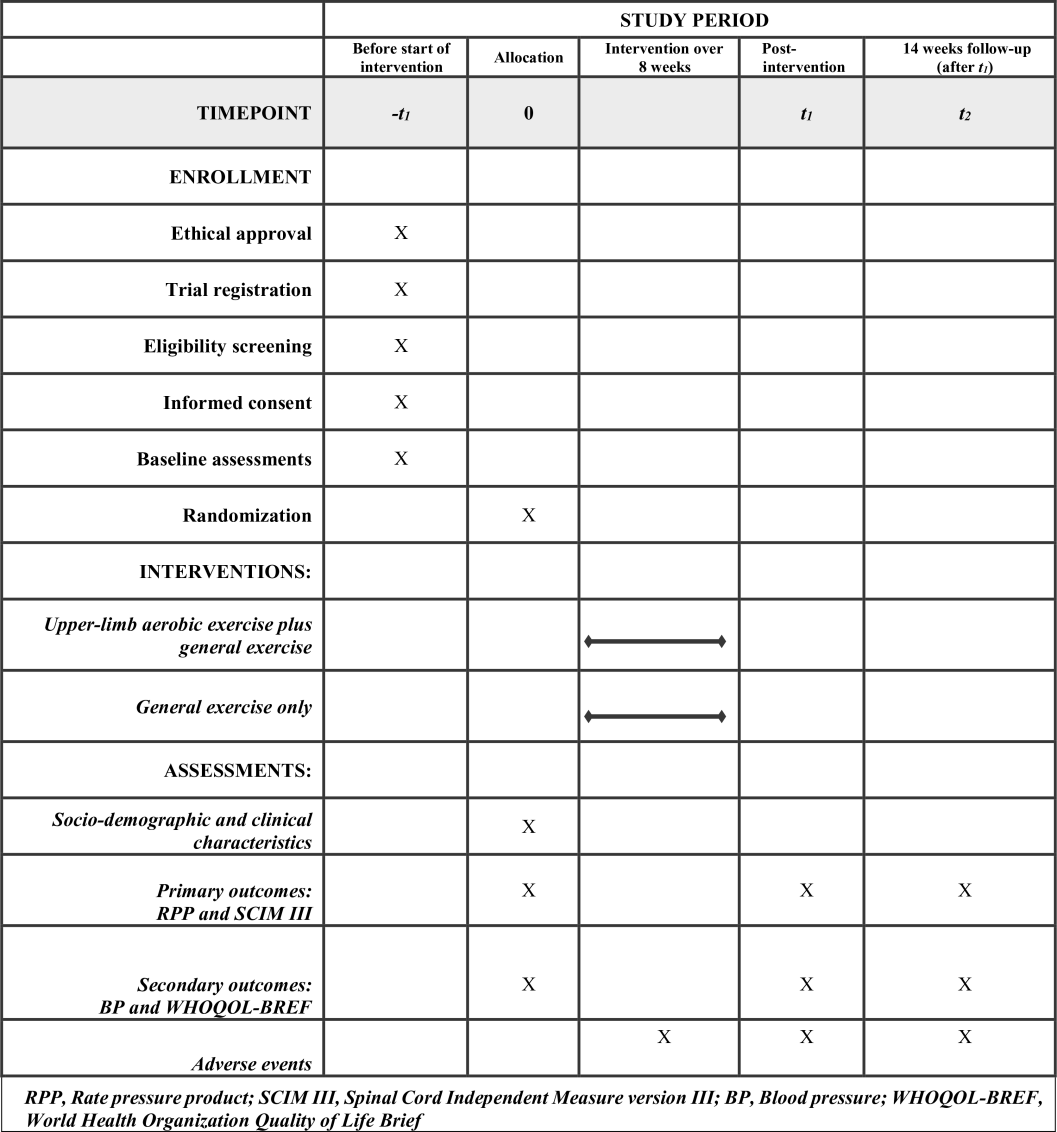
Schedule of enrollment, interventions, and assessments.

Outcome assessments will be conducted prior to randomization by a physiotherapist who will not take part in other aspects of the study (Fig 2). To enhance the consistency of self-report questionnaires, outcome assessors at each of the study centers will be trained by the primary researcher in interviewer-administered questionnaire techniques prior to data collection. Assessments will be performed at baseline (pre-intervention), 8 weeks post-intervention, and 14 weeks follow-up.

### Primary outcome measures

The primary outcomes are the RPP to be assessed using a digital BP monitor and functional disability to be assessed using the SCIM-III.

1. **Rate pressure product (RPP):** This is calculated as the product of SBP and heart rate (HR). It serves as a measure of cardiac workload, reflecting the stress put on the cardiac muscle based on the number of beats per minute. RPP is a clinically validated tool for assessing cardiac workload and cardiovascular health [21]. To measure BP (SBP and DBP) and HR, a digital BP monitor (Omron M2, HEM-7143-E) will be used. The participant will be seated comfortably with their back and arm fully supported on a flat surface at heart level. The cuff of the sphygmomanometer will be placed over the bare upper arm with the artery mark positioned directly over the brachial artery. By pressing the start button, the BP (SBP and DBP in millimeters of mercury [mmHg]) and HR in beat per minute (bpm) will be displayed on the large digital panel and recorded. The digital BP monitor is preferred because minimizes discomfort from cuff over-inflation and provides quick measurements. Intra-rater reliability of the BP measurements will be reported in the main trial.
2. **Spinal Cord Independence Measure version III (SCIM-III):** This will be used to assess the functional independence of the participants. It consists of 19 tasks organized into three subscales: self-care, respiration and sphincter management, and mobility. Each subscale is scored on a 100-point scale (self-care: 0–20; respiration and sphincter management: 0–40; mobility: 0–40), with 0 indicating total dependence and 100 indicating complete independence [22]. The SCI-III has demonstrated adequate reliability (Kappa score = 0.696–0.983; Cronbach’s alpha = 0.923) and validity [23].

### Secondary outcome measures

The secondary outcome measures include BP (SBP and DBP) and QOL. Participants’ QOL will be assessed using the World Health Organization Quality of Life Scale (WHOQOL-BREF). The WHOQOL-BREF, was developed specifically for patients with SCI to evaluate QOL and is the most widely used questionnaire [24], with adequate reliability and validity [24,25]. It consists of 26 items measuring four domains: physical health, mental health, social relationships, and environment. Each item is ranked on a 5-point Likert scale. The higher the scores the better the QOL [24]. Participants will be instructed to consider their experiences over the previous 15 days when responding to the questionnaire items.

### Data analysis

All statistical analyses will be conducted on IBM SPSS Statistics version 24.0 (IBM Co., Armonk, NY, USA), with a significance level at 0.05.

Descriptive statistics of means and standard deviations will be used to summarize continuous variables, while frequencies and percentages will be used for categorical variables. Data distribution will be assessed statistically using the Kolmogorov–Smirnov and Shapiro–Wilk tests, and visually through histograms, Q-Q plots, and box plots. Baseline comparisons of continuous variables between groups will be conducted using independent *t-*test while the chi-square (χ²) test will be used for categorical variables. For normally distributed data, a mixed model analysis of variance (ANOVA) with a 2 x 3 design (treatment group [ULAE group versus control group] x time [baseline, 8-week post-intervention, and 14-week follow-up]) will be employed to evaluate treatment effectiveness. The treatment group (between-subject factor) will be treated as a fixed effect, while time (within-subject factor) will be treated as a random effect. For non-normally distributed data, the Mann–Whitney *U* test will be used. Potential confounding variables, such as sex, education level, BMI, duration of injury, and type of injury will be adjusted for in the analysis. The intention to treat (ITT) principle will be applied, with all randomized participants included in the analysis according to their originally assigned treatment groups, regardless of whether they completed treatment.

### Data management

Participant data will be recorded in both a logbook and electronically using Microsoft Excel sheets stored on a secure computer hard drive. Prior to data entry, all data will undergo thorough error-checking procedures to ensure accuracy and completeness.

### Dissemination plans

The findings of this study will be disseminated through a knowledge-translation workshop, publication in a peer-reviewed journal, and presentation at a national or international conference.

## Discussion

The management of SCI requires considerable health care resources and can impose a significant financial burden on the affected individuals, their families, and the community [26]. This is particularly concerning in resource-constrained regions, such as northern Nigeria, where many SCI victims come from low-socioeconomic backgrounds [27] and experience low QOL [28], in addition to limited or lack of rehabilitation centers [27,29]. This study aims to determine the effects of ULAE plus general exercise program versus general exercise alone among patients with SCI in northern Nigeria. Based on our hypotheses, we anticipate that participants receiving the ULAE plus general exercise will demonstrate significantly greater improvements in RPP, functional independence, BP, and QOL compared to those receiving general exercise alone. Improvements in these outcomes are expected to help the patients return more quickly to their ADL and maintain an adequate level of physical fitness. The primary strength of this study lies in its design as an RCT with some key methodological features. Additionally, the intervention is both accessible and affordable, as the arm ergometer and reciprocal pulley can be improvised at minimal cost.

However, the study has several potential limitations worth noting. First, due to the nature of the interventions, blinding both participants and therapists will not be feasible. Second, although a formal sample size calculation was performed, the trial is powered to detect only a 10% difference in function, as measured by the SCIM III, between the groups, accounting for the anticipated high dropout rate. Third, due to a lack of funding, only a few outcomes will be evaluated in this trial. Any additional limitations observed during the study will be reported in the main trial publication.

### Trial status

The study opened to participant recruitment on 1 February 2023. The final follow-up is anticipated to be completed by 30 December 2024. The current version of the trial protocol is 6.0.

## Supporting information

S1 Appendix SPIRIT 2013 Checklist. (DOCX)

## Abbreviations:

ADL: Activities of daily living
AKTH: Aminu Kano Teaching Hospital
ANOVA: Analysis of variance
ASIA: American Spinal Injury Association Impairment Scale
BP: Blood pressure
BMI: Body mass index
DBP: Diastolic blood pressure
CVD: Cardiovascular disease
HR: Heart rate
MMSH: Murtala Mohammed Specialist Hospital
NOHD: National Orthopaedic Hospital Dala
RPP: Rate pressure product
SCIM III: Spinal Cord Independence Measure version III
SBP: Systolic blood pressure
SCI: Spinal cord injury
QOL: Quality of life
ULAE: Upper-limb aerobic exercise
WHOQOL-BREF: World Health Organization Quality of Life Scale
